# A protocol for tracking scholarly output to evaluate the impact of the RADx-UP program on community-engaged COVID-19 research

**DOI:** 10.1017/cts.2025.10138

**Published:** 2025-09-02

**Authors:** Valerie A. Lucas, Marlena L. Kuhn, Kristen D. Witkemper, Tara Carr, Abisola Osinuga Snipes, Barrie E. Hayes, Michelle Song, Leah Frerichs, Gaurav Dave

**Affiliations:** 1 Department of Epidemiology, Gillings School of Global Public Health, University of North Carolina at Chapel Hill, Chapel Hill, USA; 2 Center for Thriving Communities, School of Medicine, University of North Carolina at Chapel Hill, Chapel Hill, USA; 3 Health Sciences Library, University of North Carolina at Chapel Hill, Chapel Hill, USA; 4 Department of Health Policy and Management, Gillings School of Global Public Health, Chapel Hill, USA

**Keywords:** Publication search strategy, publication tracking, publication evaluation, coordinating center evaluation, publication portfolio tracking

## Abstract

**Introduction:**

Portfolio-level publication tracking collects research output from related programs. Tracking publications is imperative to evaluate the scholarly impact of a program, synthesize program findings, and document impact to funders. A valid tracking protocol increases data quality for accurate impact assessment, but there is little literature on publication tracking methods appropriate for assessing impact across multiple programs.

**Methods:**

We tracked, managed, and evaluated publications from the National Institutes of Health-funded Rapid Acceleration of Diagnostics - Underserved Populations, which included over 137 projects and a Coordination and Data Collection Center. During the four-year project, we deployed a quarterly self-report survey to project leads and conducted twice-monthly searches for grant-related publications. Search strategies comprised a simple search of project grant numbers and an enhanced search. We evaluated the sensitivity and positive predictive value of search strategies compared to the surveys.

**Results:**

Compared to the survey, the simple search was 21.5% to 27.4% sensitive with a positive predictive value between 81.1% and 95.8%. The enhanced search was 62.6% to 68.0% sensitive with a positive predictive value between 76.2% and 96.9%. Response rates declined over time from a maximum of 61.3% to a minimum of 32.8%.

**Conclusions:**

The enhanced search increased specificity in identifying publications, but the survey was necessary to refine strategies and identify missed products. However, the enhanced search may have relieved participant burden in entering citations. These findings may be valuable for coordinating centers, academic departments, working groups, and other academic entities that must quantify the impact of their publications.

## Introduction

The Rapid Acceleration of Diagnostics - Underserved Populations (RADx-UP) program sought to understand and increase access to COVID-19 testing and vaccination among communities that have been underserved or marginalized. RADx-UP supported projects to implement community-engaged research throughout the United States, its territories, and Tribal Nations. The National Institutes of Health (NIH) funded 137 RADx-UP projects [1–3] and a Coordination and Data Collection Center (CDCC) [4] to further program goals. The CDCC provided technical and administrative support to projects, managed the RADx-UP data repository, facilitated COVID-19 testing procurement, and evaluated program outcomes and impacts [5]. Understanding the impact of research from RADx-UP projects was one of the key evaluation objectives of the program, particularly since RADx-UP worked with communities that have historically been excluded or underrepresented in medical research and experience a disproportionate impact of COVID-19 [6]. Identifying RADx-UP research products was foundational in evaluating the scientific and community impact of RADx-UP on COVID-19 detection, prevention, and treatment for underserved and vulnerable populations [6].

Basic methodological guidance regarding publication tracking is available from select university library websites [7,8], but detailed processes by which research programs can accurately track resultant publications are insufficiently defined in available scholarly literature. Well-established publication tracking methods are particularly needed for large research programs comprised of multiple grants, projects, and investigators to demonstrate return on investment; these may include programs funded through NIHU24 Cooperative Agreements, Clinical and Translational Science Awards (CTSA), or non-governmental organizations [9]. U24 awards facilitate the sharing of resources and expertise across project teams and have substantially increased in quantity (93 versus 498 awards issued) and magnitude ($140,293,888 versus $894,256,147 in total funding) between 2014 and 2023 [10]. CTSA hubs receive funding through the National Center for Advancing Translational Sciences (NCATS) to bolster inter-institutional research collaborations and facilitate translation of basic science research to medical and public health practice [11]. These hubs can employ bibliometrics, social network analysis, and topic analysis to demonstrate return on investment through increased research productivity, strengthened research collaborations, accelerated translation of research to clinical practice, and alignment of research topics with hub goals and priorities [12,13]. Non-governmental research funders can apply similar methods to evaluate the impact of philanthropic research programs in furthering knowledge and changing clinical practice [14]. A complete and verified collection of research outputs increases the accuracy of program evaluation, whether assessing citation impact through bibliometrics, patterns of scientific collaboration through social network analysis, or summarizing the research findings themselves through topic analysis.

The first objective of this study was to track, organize, verify, and report publications from the RADx-UP consortium. The second objective was to obtain, organize, and report conference presentations, additional grants, patents, and non-scholarly products from the RADx-UP consortium. This study adds to the literature on the pros and cons of a variety of methods for tracking research products to improve evaluation of research programs.

## Materials and Methods

### Pilot Phase

To develop publication tracking methods, the evaluation team met with NIH leadership and CDCC project staff to understand the need for tracking and evaluation and explored methods and sources for data collection. As part of the coordinating center, we did not have access to the Research Performance Progress Reports (RPPRs) of individual RADx-UP projects. We explored using publicly available data in NIH RePORTER, but this tool often includes publications from a parent grant, which exaggerates the number of publications attributable to the specific RADx-UP project. Further, data from NIH RePORTER often were not updated in the timeframe needed for RADx-UP publication analyses. After concluding that we would need to develop an independent system to track publications, we coordinated with the RADx-UP CDCC Publications & Dissemination Committee to ensure tracking efforts were not duplicated across the center and develop language on how to acknowledge the RADx-UP program in publications for committee policies. Projects were encouraged to use the following language when publishing an article: “National Institutes of Health (NIH) support was provided in part by grants from [Institute or Center Name]. [Grant or contract #] as a part of the RADx-UP program. [Project-specific acknowledgment statement (acknowledging project, staff, contributions of participants, etc.)].” We also developed a one-page brief to further disseminate the importance of publication tracking, and thus the importance of clear program acknowledgments, to project staff.

**Figure 1.**
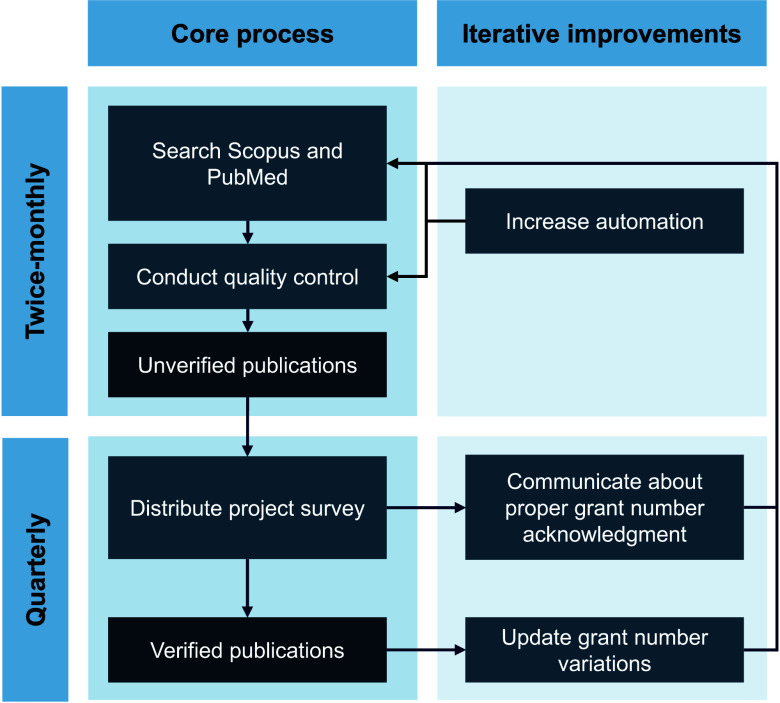
Diagram depicting the processes and improvements involved in tracking publications from the RADx-UP program using database searches and project surveys.

On August 1, 2021, we distributed a pilot survey in Qualtrics v07.2021 [15] to RADx-UP project leads to collect data on both pending and published scholarly articles, conference presentations or abstracts, and intramural and extramural grants associated with their projects. In some publications submitted, we observed an absence of grant number acknowledgments or inconsistency in the formatting of grant numbers. For example, the grant number “3U24MD016258-01” might be cited as “3U24-MD016258-01,” “3 U24 MD016258-01,” or “U24-MD016258-01.” Incorporating lessons learned from pilot testing and feedback from partners, we developed a system to track publications from all 137 RADx-UP projects by searching for RADx-UP project grant numbers and 8 additional variations per grant number in Scopus and PubMed databases on the first and fifteenth of each month and surveying RADx-UP project leads every three months (Figure 1).

### Database searches

Data collection from Scopus and PubMed databases occurred twice per month from October 15, 2021 to January 15, 2025. Initially, we used just one spelling variation of the grant number: the full number without spaces. However, this “simple search” failed to detect many publications due to variation in how authors spelled the grant numbers. Authors may omit leading numbers and letters or add hyphens and spaces to grant numbers in the funding section. To capture these variations, we established an “enhanced search including a programming script in Python v3.9.7 [16] to generate spelling variations of each RADx-UP grant number (see supplementary materials 1 and 2 for script). As we identified more publications, we updated the script to capture additional spelling variations. This program converted the augmented grant number list into database search strings to identify RADx-UP grant numbers within the funding text of publications. Each quarter, we reviewed the funding text of publications reported in the survey, noted any new variations in grant numbers reported, and added variations to the enhanced search. The script is available as a supplement in the online version of this article.

We processed search results using a programming script in R version 4.1.0 [17] to merge new data with existing publications, deduplicate publications (based on Digital Object Identifiers [DOIs], PubMed IDs, and titles), and assign tags to articles. Article tags included 1) RADx-UP project(s) associated with the article based on either grant number acknowledgment or survey responses; 2) whether a RADx-UP project lead verified the publication in the survey; 3) whether a RADx-UP project lead confirmed or rejected the publication in the survey; 4) whether the publication acknowledged NIH in the acknowledgments or funding section; 5) whether the publication acknowledged the RADx-UP program name in the acknowledgments or funding section; 6) whether the publication correctly acknowledged the RADx-UP grant number; and 7) whether the title or abstract mentioned COVID-19 or community engagement. We automatically assigned tags during processing but manually verified results to correct errors caused by incorrect indexing or missing information in funding text and acknowledgments sections in search results. Next, we exported the processed search results to a shared Excel file, with one row representing a particular publication for a particular project. At the end of the publications tracking initiative, we analyzed the sensitivity and positive predictive value (PPV) of this strategy in identifying publications by comparing search results using different versions of grant numbers (enhanced search) to search results using only the original grant numbers (simple search).

### Project surveys

We distributed quarterly surveys to RADx-UP project leads from August 1, 2022 to November 30, 2024 (Supplementary material 3 for survey). Study data were collected and managed using Research Electronic Data Capture (REDCap) hosted at UNC [18,19]. REDCap is a secure, web-based software platform designed to support data capture for research studies. Project leads received a survey link via email on the first of each month after we processed database search results. We imported the citations of search results from the previous quarter into the surveys for project leads to either confirm or reject the publication’s relation to their RADx-UP work. Project staff could also view a list of their previously reported products in the survey and report on any scholarly publications that were not identified through the database searches, entering the title, author list, journal, date, and DOI. Projects had three weeks to complete the survey and received weekly reminders, with extensions granted upon request. Additional publications reported were reviewed before each database search and DOIs for these articles were added to the search strings once available. Staff additionally reported on conference presentations, additional grants, patents, and non-scholarly products associated with their RADx-UP project, providing the title, date, conference or funder name, and link if applicable.

Since the evaluation team surveyed projects and asked them to report their publicly available products, the UNC Institutional Review Board (IRB) determined that the publication tracking project was Not Human Subjects Research.

## Results

**Figure 2.**
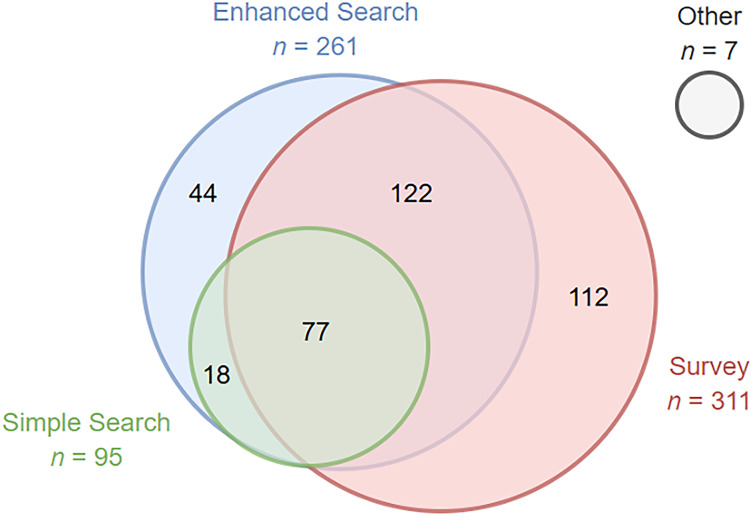
Venn Diagram illustrating the number of publications from the RADx-UP program identified using various publication tracking methods. The simple search included a single grant number spelling per project, the enhanced search included additional variations of grant number spellings per project, and the survey enabled projects to report additional publications that were not identified from the searches. Other publications were reported by CDCC staff.

We identified 414 publications from the RADx-UP program using the publication tracking system. We restricted analyses to 380 publications related to COVID-19 or community engagement to retain topical relevance to RADx-UP. As of January 15, 2025, the simple search identified 95 publications, the enhanced search identified 261 publications, and the REDCap survey identified 311 publications (Figure 2). CDCC staff also independently reported seven publications that were never identified using the searches or surveys. Project leads rejected four publications from the simple search and eight from the enhanced search. Due to project non-response, 14 publications from the simple search were never verified and 54 publications from the enhanced search were never verified.

**Figure 3.**
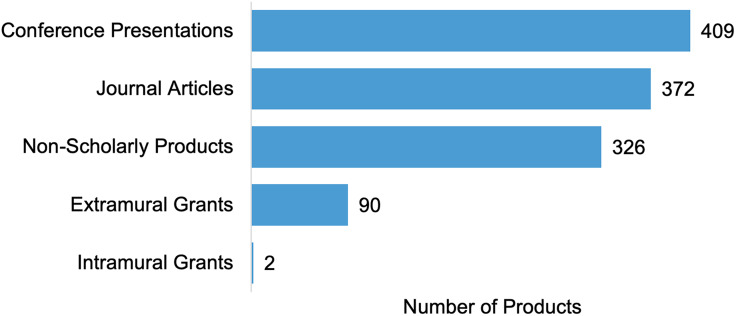
Types of products reported by project leads from the RADx-UP program. Journal articles include publications that were related to COVID-19 and were confirmed or unverified by project staff, excluding the eight publications that project staff rejected.

**Figure 4.**
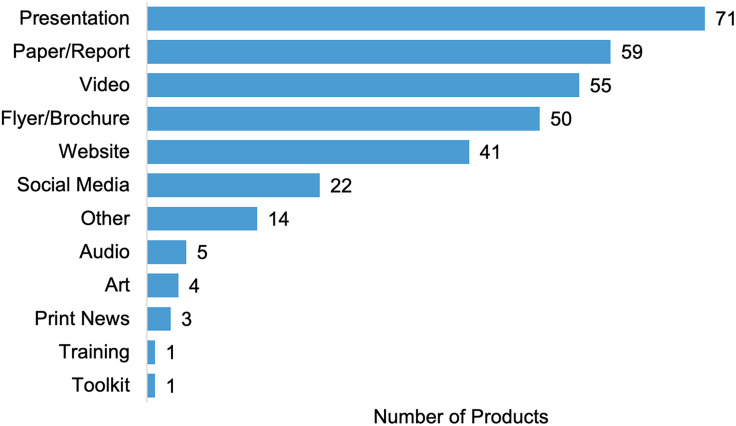
Types of community or non-scholarly products reported by project leads from the RADx-UP program.

Projects also identified products not available in Scopus or PubMed through the surveys, including 409 conference presentations and 92 additional grants awarded to RADx-UP projects (Figure 3). Additionally, projects reported 326 community or non-scholarly products, with the most frequent products being presentations, reports, and videos (Figure 4). If a public link to the product was available, we forwarded the product to CDCC staff who curated the Community Engagement Library. The library reviewed and published community-related materials to the RADx-UP public website. Conference presentations and additional grants were included to capture dissemination of RADx-UP research to the larger research community and continuation or expansion of research activities. Non-scholarly products were included to capture sharing of findings with the broader public. However, evaluating the impact of these products was challenging with the data reported and these products are not indexed in the databases we had access to.

Using the survey as the standard, we calculated the bounds of sensitivity and positive predictive value (PPV) based on logical possibilities for the 54 unverified publications from the enhanced search. We quantified the “worst case” where all unverified publications found in the search would have been rejected and all unverified publications not found in the search would have been confirmed, and the “best case” where all unverified publications found in the search would have been confirmed and all unverified publications not found in the search would have been rejected (Table 1). The sensitivity of the simple search ranged from 21.5% in the worst-case scenario to 27.4% in the best-case scenario, and the sensitivity of the enhanced search ranged from 62.6% in the worst-case scenario to 68.0% in the best-case scenario. The PPV of the simple search ranged from 81.1% in the worst-case scenario to 95.8% in the best-case scenario, and the PPV of the enhanced search ranged from 76.2% in the worst-case scenario to 96.9% in the best-case scenario. Table 2 summarizes the pros and cons of each tracking method.

**Table 1.** Number of publications from the RADx-UP program identified through the simple search and enhanced search. The simple search included a single grant number spelling per project, while the enhanced search included additional variations of grant number spellings per project. Publications are stratified by whether the publications were confirmed or rejected by project staff in the survey. In the “worst case,” all unverified publications found in the search would have been rejected and all unverified publications not found in the search would have been confirmed. In the “best case” all unverified publications found in the search would have been confirmed and all unverified publications not found in the search would have been rejected. Only publications that were found in the enhanced search could be presented to project staff in the survey for confirmation or rejection

	Worst case		Best case	
	Confirmed	Rejected	Confirmed	Rejected
*Simple search*				
Found in search strategy	77	18	91	4
Not found in search strategy	281	4	241	44
	Sensitivity 21.5%	PPV 81.1%	Sensitivity 27.4%	PPV 95.8%
*Enhanced search*				
Found in search strategy	199	62	253	8
Not found in search strategy	119		119	
	Sensitivity 62.6%	PPV 76.2%	Sensitivity 68.0%	PPV 96.9%

**Table 2.** Summary of pros and cons for each publication tracking method

	Pro	Con
Simple search	No participation from projects required if grant numbers are known Easy to implement compared to the enhanced search and survey	Misses more publications compared to both enhanced search and survey
Enhanced search	No participation from projects required if grant numbers and their variations are known Identifies more publications than the simple search	More complex to implement than the simple search Requires prior knowledge of variations in grant number spellings, which may be specific to projects Identifies less publications than the survey Publications identified using the grant number may be rejected by project teams in the survey
Survey	Identifies the most publications Allows for collection of conference presentations, grants, and non-scholarly products	Requires participation from projects Requires development and management of surveys

**Figure 5.**
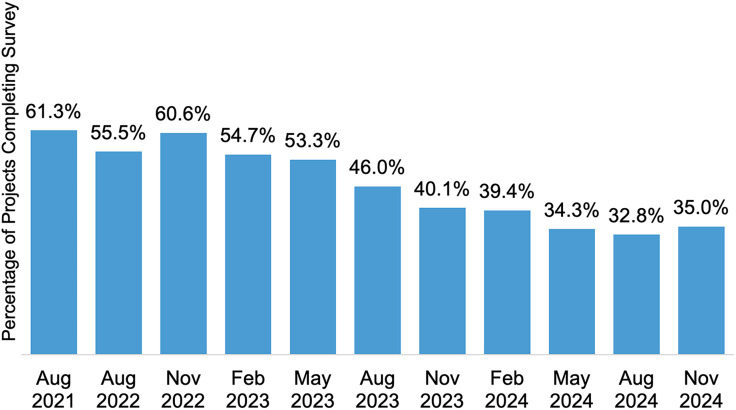
Percentage of projects (*N* = 137) from the RADx-UP program that completed the survey to report scholarly and non-scholarly products by timepoint.

**Figure 6.**
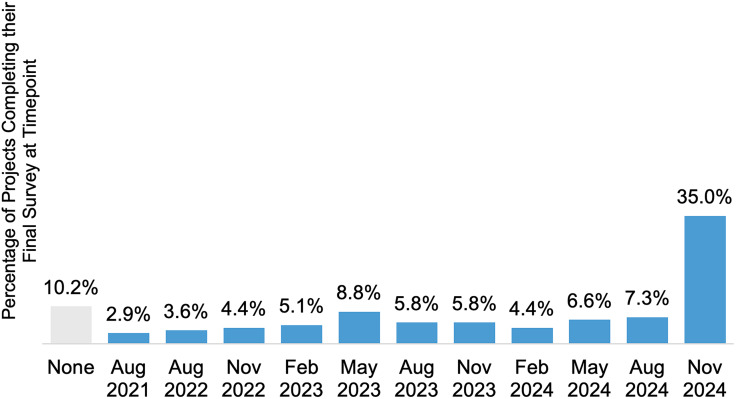
Percentage of projects (*N* = 137) from the RADx-UP program that completed their last survey to report scholarly and non-scholarly products at each timepoint. Projects indicated in gray never completed a survey at any timepoint.

The survey received the most project response in the pilot phase, with 61.3% of project teams responding to the survey, followed closely by 60.6% in the second timepoint after the pilot phase (Figure 5). By the last timepoint, project response rates were nearly halved at 35.0%, with 48 project teams responding. Other projects dropped out steadily throughout data collection and 10.2% of projects never responded to the survey at any timepoint (Figure 6).

## Discussion

This study contributes to the limited literature on the utility of various methods for tracking scholarly output from large research consortia to enable a more comprehensive evaluation of their research impact. Our results indicate that an enhanced search for grant number variations in publication databases can increase sensitivity in identifying a portfolio of publications from research consortia while maintaining high positive predictive value compared to a strategy that searches for a single grant number spelling. However, projects themselves remain an important data source, reporting nearly 30% of publications that the searches would have otherwise missed. Furthermore, 10.8% of articles in the survey correctly acknowledged the grant number but were never identified in the searches. In addition to missing publications due to irregular or missing grant number citations, the enhanced search could not identify articles if they were not indexed in the databases or if their funding text was incorrectly indexed. Additionally, the enhanced search can identify spurious results when authors disclose funding from RADx-UP in publications unrelated to the project. Although the RADx-UP grant period concluded in June 2025, the enhanced search could have been improved by using other program information that could be acknowledged in publications in addition to or in the absence of grant numbers, such as variations of the RADx-UP project name, individual project names, names of community organization partners, names of project staff, or project staff authorship. For example, 31.5% of articles reported in the surveys mentioned RADx-UP in the acknowledgements. Future publication tracking methods should consider all available program information to overcome limitations in searches based on project grant numbers.

The survey was also necessary to capture additional products such as grants or non-scholarly products that are not well-indexed in databases. However, products reported in survey responses were often not sufficiently organized or accessible to facilitate data use beyond basic counts. Other tools, such as Dimensions [20], may be useful for collecting standardized data on other types of products but are costly. Better tracking and evaluation of publications should lead evaluators to go beyond scholarly published work when evaluating research dissemination. Beyond publications, researchers may create presentations, workshops, study briefs, or social media communications to engage a broader audience. Accessible dissemination to community partners is critical to building trust in public health research, and the survey allowed for wider distribution of curated community engagement research products beyond the projects themselves in the CDCC Community Engagement Library.

There were strengths and limitations to each approach, but the survey was the most comprehensive, identifying the most publications and producing a high confidence dataset with input from RADx-UP projects. Quarterly surveys helped to maintain the cadence of receiving reliable information about publications. However, there were several drawbacks to using a survey. Response rates decreased throughout data collection, suggesting respondent burden and limiting our ability to confirm search-identified publications. We attempted to alleviate this burden by presenting the projects with publications identified through the search strategy for verification. However, lower response rates created additional uncertainty in the results as project non-responses led publications from the searches to remain unverified and publications with irregular grant acknowledgements to be missed. Publication tracking was also time-consuming for the staff implementing the process, often requiring manual input. Although programming scripts supported partial automation of the process, additional resources, such as time, staff, and software, could have facilitated further automation.

The duration of data collection was also a limitation. Publication tracking ended alongside funding of the CDCC, but the publication process can be long, with delays in the submission, peer review, and resubmission processes. Research teams may continue to work on publications well past the official end of a project. Additionally, researchers may use study data for future secondary analyses. Future publication tracking projects can consider adapting our approach to their specific project needs and capacities over a longer timeframe to increase their ability to identify publications from large research consortia without substantial increases in effort. Clear instructions for authors about how to format grant numbers when publishing may improve detection of publications through the simple and enhanced search. Additionally, the ability to access grant reports could improve and reduce the need for a survey.

Researchers, funders, and other stakeholders can track publications using a variety of methods to demonstrate return on investment for research programs through evaluation methods such as bibliometric analysis, network analysis, and topic analysis. These analyses can quantify the impact of a research program’s scholarship, identify relationships among collaborators in the program, and demonstrate productivity on specific topics. The tracking and validation process is the first step in showing impact by identifying accurate inputs for analysis and interpretation. Evaluators must thoughtfully consider the pros and cons of methodologies for tracking scholarly output within the research context of interest to select and implement effective methods for collecting data on research outputs.

## Supporting information

10.1017/cts.2025.10138.sm001 Lucas et al. supplementary material 1 Lucas et al. supplementary material

10.1017/cts.2025.10138.sm002 Lucas et al. supplementary material 2 Lucas et al. supplementary material

10.1017/cts.2025.10138.sm003 Lucas et al. supplementary material 3 Lucas et al. supplementary material

## Supplementary material

The supplementary material for this article can be found at https://doi.org/10.1017/cts.2025.10138.
