# Supplementary material for: A protocol for tracking scholarly output to evaluate the impact of the RADx-UP program on community-engaged COVID-19 research
Source: J Clin Transl Sci. 2025 Sep 2;9(1):e219. doi: 10.1017/cts.2025.10138 (PMC12529626; doi:10.1017/cts.2025.10138)
Supplement: Lucas et al. supplementary material 3 — Lucas et al. supplementary material [file S2059866125101386sup003.pdf]

Scholarly & Non-scholarly Product Survey

Project [Project Number]: [Project Title]

Survey Purpose

The Tracking & Evaluation team is interested in collecting information about the scholarly and non-scholarly products associated with your RADx-UP project to evaluate the **research impact** of RADx-UP and the **strength of research collaborations** within RADx-UP.

Our team **will not** be comparing the research impact of individual RADx-UP projects. Instead, we will analyze the overall research impact of RADx-UP by **aggregating data on scholarly and non-scholarly products across projects**. The Tracking & Evaluation team will share de-identified data and visualizations with the NIH. Your responses also help populate the **RADx-UP publications dashboard** and may be displayed in the **RADx-UP Engagement Resource Library** with your consent.

Our definition of scholarly and non-scholarly products include other products, grants or fellowships directly **associated with your RADx-UP project,** which means the product is supported by the RADx-UP grant. We are no longer asking for *pending* products.

Survey Length

If you have no additions or updates to the list of scholarly and non-scholarly products on file for your RADx-UP project, the survey will take about **2 minutes** to complete. If you have many changes to the list on file, the survey could take **15-20 minutes** to complete.

The survey has a **Save and Return Later** feature turned on so that you can complete the survey in multiple settings via the same link. The link is **unique to your project** and can be forwarded to other members of your team for assistance in completion.

Please contact [Name] at [Email] if you have any questions or comments.

Indicate the type(s) of scholarly & non-scholarly products that your project has produced.

- [x] **Journal articles**
  Articles published in a peer-reviewed academic journal
- [x] **Conference presentations/abstracts**
  Conference presentations or abstracts in an academic conference

- [x] **Additional grants and other funding**
  Additional grants, fellowships, or other funding types continuing the activities of the RADx-UP grant
- [x] **Patents**
  Intellectual property filings with the U.S. Patent Office
- [x] **Non-scholarly products**
  Communication products (including presentations, websites, videos, art etc.) not part of an academic journal or conference
- [ ] None of the above

Journal Articles

New or Updated Journal Articles

Please **add** or **update** published journal article(s) **associated with your RADx-UP project**.

Publication

**Author(s)**	Exampleton A.B., Exampleson C.D., Exampleron E.F.
**Article Title**	Show me the way: examples clarify concepts
**Journal**	Example journal
**Publication Date**	07-01-2021
**DOI**	10.4103/0976-500X.110894
**Author(s)**	
**Article Title**	
**Journal**	
**Publication Date**	M-D-Y
**DOI**	

**Author(s)**	
**Article Title**	
**Journal**	
**Submission Date**	M-D-Y
**DOI**	

Author(s)
Article Title
Journal
Publication Date M-D-Y
DOI

Author(s)
Article Title
Journal
Publication Date M-D-Y
DOI

Author(s)
Article Title
Journal
Publication Date M-D-Y
DOI

☐ I need more space to add or update journal articles that are associated with my RADx-UP project.

Conference Presentations/Abstracts

New or Updated Conference Presentations/Abstracts

Please **add** or **update** presented conference presentations/abstracts **associated with your RADx-UP project**.

Presentation or Abstract

**Author(s)**	Exampler Y.Z., Examples W.V., Examplet U.T.
**Presentation Title**	Examples: Demonstrating, showing, illustrating
**Conference**	Sample Conference
**Presentation Date**	07-01-2021
**Link**	RADx.UP.org

Author(s)
Presentation Title

Conference

Presentation Date M-D-Y

Link

Author(s)

Presentation Title

Conference

Presentation Date M-D-Y

Link

Author(s)

Presentation Title

Conference

Presentation Date M-D-Y

Link

Author(s)

Presentation Title

Conference

Presentation Date M-D-Y

Link

Author(s)

Presentation Title

Conference

Presentation Date M-D-Y

Link

☐ I need more space to add or update conference presentations/abstracts that are associated with my RADx-UP project.

Additional Grants and Funding Sources

New or Updated Additional Grants and Funding Sources

Please **add** or **update** additional grants and funding sources **associated with your RADx-UP project** that have been awarded.

Grant or Fellowship

Intramural/ Extramural Extramural

**Funding Institution**	Foundation for Clear Examples
**Grant Number**	ABC123XY-Z890
**Funding Amount**	$10,000
**Funding Start Date**	07-01-2021
**Funding End Date**	07-01-2022
**Grant Title**	The Example Fund: Clarifying concepts worldwide
**Intramural/ Extramural**	
**Funding Institution**	
**Grant Number**	
**Funding Amount**	$
**Funding Start Date**	M-D-Y
**Funding End Date**	M-D-Y
**Grant Title**	
**Intramural/ Extramural**	
**Funding Institution**	
**Grant Number**	
**Funding Amount**	$
**Funding Start Date**	M-D-Y
**Funding End Date**	M-D-Y
**Grant Title**	
**Intramural/ Extramural**	
**Funding Institution**	
**Grant Number**	
**Funding Amount**	$
**Funding Start Date**	M-D-Y
**Funding End Date**	M-D-Y
**Grant Title**	
**Intramural/ Extramural**	
**Funding Institution**	
**Grant Number**	
**Funding Amount**	$
**Funding Start Date**	M-D-Y
**Funding End Date**	M-D-Y

**Grant Title**	
**Intramural/ Extramural**	
**Funding Institution**	
**Grant Number**	
**Funding Amount**	$
**Funding Start Date**	M-D-Y
**Funding End Date**	M-D-Y
**Grant Title**	

☐ I need more space to add grants or fellowships that are associated with my RADx-UP project.

Patents

New or Updated Patents

Please **add** or **update** the following information about patent(s) **associated with your RADx-UP project**.

The *description* should begin by setting out the scope of application of your invention and explaining the particular technical problem that it solves. Indicate also **what the solution is**.

**Patent**	
**Status**	Pending
**Patent Title**	Example-o-matic
**Patent Holder**	Examplan E.F. **OR** UNC Center for Example Science
**Patent Number**	QWERTY-0987-1234
**Application Date**	07-01-2021
**Approval Date**	10-01-2021
**Description**	Example-o-matic solves the problem that occurs when a survey designer fails to adequately define a survey question. The Example-o-matic clarifies fields of information in surveys.
**Status**	
**Patent Title**	
**Patent Holder**	
**Patent Number**	
**Application Date**	M-D-Y
**Approval Date**	M-D-Y

Description

100 words remaining

Status

Patent Title

Patent Holder

Patent Number

Application Date M-D-Y

Approval Date M-D-Y

Description

100 words remaining

Status

Patent Title

Patent Holder

Patent Number

Application Date M-D-Y

Approval Date M-D-Y

Description

100 words remaining

Status

Patent Title

Patent Holder

Patent Number

Application Date M-D-Y

Approval Date M-D-Y

**Description**	
	100 words remaining
**Status**	
**Patent Title**	
**Patent Holder**	
**Patent Number**	
**Application Date**	M-D-Y
**Approval Date**	M-D-Y
**Description**	
	100 words remaining

☐ I need more space to add patents that are associated with my RADx-UP project.

Non-Scholarly Products

New or Updated Non-Scholarly Products

Please **add** or **update** non-scholarly product(s) **associated with your RADx-UP project**.

The *description* should include information about the content, purpose, intended audience, and publishing platform.

**Product**	
**Title**	The ExampleCast
**Author/Creator**	Exampleson E., Exemplary B.C.
**Date**	01-26-2022
**Type**	Audio
**Description**	Engages listeners with examples to clarify survey questions; published on Spotify and Apple podcasts for survey takers.
**Link**	RADx.UP.org
**Title**	
**Author/Creator**	
**Date**	M-D-Y

Type

Description

100 words remaining

Link

Title

Author/Creator

Date M-D-Y

Type

Description

100 words remaining

Link

Title

Author/Creator

Date M-D-Y

Type

Description

100 words remaining

Link

Title

Author/Creator

Date M-D-Y

Type

Description

100 words remaining

Link

Title

Author/Creator

Date M-D-Y

Type

Description

100 words remaining

Link

☐ I need more space to add published products that are associated with my RADx-UP project.

Additional Comment

Please provide any additional comments about your entries above if needed.

Submit

Save & Return Later

Powered by REDCap - Cookie policy
